# Sunscreen lotions in the dermatological prescription: review of concepts and controversies^☆^

**DOI:** 10.1016/j.abd.2021.05.012

**Published:** 2022-01-14

**Authors:** Flavia Alvim Sant'anna Addor, Carlos Baptista Barcaui, Elimar Elias Gomes, Omar Lupi, Carolina Reato Marçon, Hélio Amante Miot

**Affiliations:** a Grupo MEDCIN (Centro de Pesquisa Clínica), São Paulo, SP, Brazil; b Dermatology Service, Faculdade de Ciências Médicas, Universidade do Estado do Rio de Janeiro, Rio de Janeiro, RJ, Brazil; c Cancer Center, Beneficência Portuguesa de São Paulo, São Paulo, SP, Brazil; d Dermatology Service, Universidade Federal do Estado do Rio de Janeiro, Rio de Janeiro, RJ, Brazil; e Policlínica Geral do Rio de Janeiro, Rio de Janeiro, RJ, Brazil; f Clinical Immunology Service, Universidade Federal do Rio de Janeiro, Rio de Janeiro, RJ, Brazil; g Dermatology Service, Santa Casa de Misericórdia de São Paulo, São Paulo, SP, Brazil; h Dermatology Department, Faculty of Medicine, Universidade Estadual Paulista, Botucatu, SP, Brazil

**Keywords:** Dermatology, DNA damage, Skin neoplasms, Solar radiation, Sun protection factor, Sunscreening agents, Ultraviolet rays

## Abstract

The skin is regularly exposed to several environmental aggressions, including solar radiation, whose biological effects can induce sunburn, dyschromia, skin aging and cancer. Among the photoprotection measures, sunscreens comprise a relevant part of the strategy aimed to prevent solar radiation damage and, for effective action, the patient must adhere to the product use and the latter, in turn, must follow technical parameters to promote adequate protection. This review article brings together the most current and relevant concepts about photoprotection for dermatological use, including the challenges for their formulation, the risks of certain photoprotective active substances for individual and environmental safety and the importance of stringency in determining the product efficacy, considering the regulatory aspects, highlighting relevant differences between Brazil and other countries. Thus, when assessing a sunscreen, not only the visual aspects and sensory perception will be immediately evaluated, but also the quality and suitability of the vehicle, the chemical composition of the formulation, the environmental risks, the photostability of the screening system, and the measurement of its protection spectrum. Technical knowledge of sunscreens can help dermatologists in this important role of educating patients about the best photoprotective strategies in each situation.

## Introduction

The skin is the main interface between the body and the environment, being exposed to oxidants, including solar radiation and environmental pollutants, such as internal combustion engine gases, cigarette smoke, halogenated hydrocarbons, heavy metals and ozone, making it the first defense system against the external environment.^1^

The solar spectrum contains ultraviolet radiation between the wavelengths of 100 to 400 nm.^2^ The solar ultraviolet (UV) radiation that reaches Earth is a combination of UVB (280–315 nm) and UVA (315–400 nm) wavelengths.^2^ UVC radiation (100–280 nm) is of no clinical significance, as it is blocked by the ozone layer and cannot reach the earth's surface.^2^, ^3^ On the skin, the acute effects of ultraviolet (UV) radiation include erythema, sunburns and photoimmunosuppression, whereas the chronic effects include photoaging and skin cancer.^2^, ^4^ Sunscreen use, along with seeking shaded areas and wearing protective clothing, hats, and sunglasses, can be very effective in the prevention of photodamage and photodermatoses caused by UV radiation.^3^, ^4^

The recommendations to mitigate the adverse effects of solar radiation, generally based on booklets and studies carried out mainly in the United States of America (USA) and Europe, are not always adequate to the Brazilian reality,^2^, ^3^, ^5^ as Brazil has one of the highest rates of UV radiation worldwide, especially in clear sky conditions.^2^, ^5^ Due to the Brazilian climate characteristics, an unprotected person can receive a daily dose of UV more than 50-fold higher than 108 J/m^2^, which is the World Health Organization (WHO) recommended maximum dose.^2^, ^5^ Access to and knowledge about the distribution of the UV index in Brazil are important subsidies for attenuating the problem of skin cancer since high doses of accumulated UV radiation are associated with the development of skin carcinomas.^5^

Among the strategies to reduce the adverse effects of the sun, the use of a broad-spectrum sunscreen is recommended, that is, one that protects against UVB and UVA radiation.^2^, ^3^, ^4^, ^6^ Conceptually, in Brazil, topical sunscreen is a cosmetic preparation applied to the skin, consisting of substances that absorb, disperse, or reflect UVB and UVA radiation.^2^, ^7^ The main purpose of sunscreen is to protect the skin from ultraviolet radiation in order to effectively minimize the damage caused by sun exposure and, concomitantly, the product must be safe, have good skin tolerability, and offer pleasant sensory properties.^3^, ^7^ In addition to sunscreens, other dermatological cosmetics can offer protection against ultraviolet UV radiation as an additional benefit. As this is not the main purpose of the product they are called multifunctional products and are submitted to a less strict technical regulation, mainly in terms of protection against UVA radiation.^7^

The prescription of sunscreens, as well as the correct orientation to patients about photoprotective measures, is part of dermatologists' routine and can be a differential to guarantee more efficient results^2^ and to prevent the previously related acute and chronic effects.

Considering the current issues that have an impact on the adequate selection of a sunscreen, the aim of this article is to provide an overview of the relevance of topical photoprotection, as well as the challenges in sunscreen formulation and the importance of stringency in determining the effectiveness of each product, considering the regulatory aspects.

## Biological effects of solar radiation on the skin

There is a growing body of observational and experimental evidence that regular exposure to solar radiation has general health benefits, both psychological and physical.^5^, ^8^ On the other hand, UV radiation is responsible for several important photochemical and photobiological reactions, capable of causing damage of varying proportions to the skin, depending on the duration of exposure, seasonal differences regarding the intensity of sunlight incidence, geographic location, and characteristics of the individual, such as age, color, and skin type (phototype), behavioral and immunological factors.^2^ Long-term effects of UV radiation exposure include premature skin aging, inhibition of the immune system, and increased risk for basal cell carcinoma, squamous cell carcinoma, and melanoma.^2^, ^9^, ^10^

In the short term, UVB exposure is responsible for sunburns (inflammation) and direct DNA damage that leads to the formation of cyclobutane pyrimidine dimers (CPDs) and 6-4 pyrimidine-pyrimidone (6-4PP) photoproducts.^9^, ^11^ While UVB radiation higher levels occur from 10 am to 3 pm, UVA radiation is more constant during the day, with elevated levels from 8 am to 5 pm.^5^ As it does not induce immediate erythema, there is no perception of UVA overdose. The initial effects of cutaneous exposure to UVA radiation are shown as the immediate pigmentary darkening of the skin and, later, in tanning. Even though UVA radiation is less energetic than UVB, it can cause mutations and cancer through an indirect mechanism involving the formation of free radicals.^11^ UVA penetrates deeper into the skin than UVB and produces reactive oxygen species (ROS) that damage the DNA, vessels, and elastic fibers in connective tissue, contributing to photoaging.^2^, ^9^, ^11^ Additionally, UVA radiation is the main concomitant cause of skin photosensitization and phototoxicity.^3^ Based on photobiological interactions with DNA, UVA radiation is often divided into UVA2 or short UVA (315–340 nm) and UVA1 or long UVA (340–400 nm).^2^, ^12^ UVA1 radiation induces epidermal and dermal damage, alterations in gene and protein expression of essential biological pathways, generation of ROS, in addition to the induction of lasting hyperpigmentation, even in individuals with highly pigmented skin.^12^, ^13^

Skin melanin pigmentation is determined by multiple factors, including the number and metabolic activity of melanocytes in the basal layer of the epidermis, the melanogenic activity of melanosomes inside these melanocytes, and variations in the number, size, and distribution of melanosomes. Melanocytes are radiation sensor cells and capture radiation to produce melanin, which can be of two different types: eumelanin (color varying from dark brown to black) and pheomelanin (color varying from yellow to red). Overall, eumelanin provides protection against solar radiation and DNA damage induced by oxidative stress, limiting the extent of UV penetration in the epidermis and eliminating ROS so that dark-skinned individuals have a lower frequency of skin cancer than fair-skinned individuals. On the other hand, pheomelanin is pro-oxidative and, upon UV radiation induction, it produces free radicals and hyperenergetic derivatives that can damage cells.^14^ Therefore, the photoprotection provided by melanin is not complete, and even dark-skinned people can also have DNA damage induced by solar radiation, although damage can be reversed by cell DNA repair mechanisms, thus reducing the risk of malignant transformation. On the other hand, in fair-skinned individuals, where pheomelanin is predominant, melanin is not sufficient to provide effective protection against solar radiation, and the extent of DNA damage may exceed the capacity of the repair mechanisms, with a higher risk of malignant transformation.^9^, ^14^

**Figure 1:**
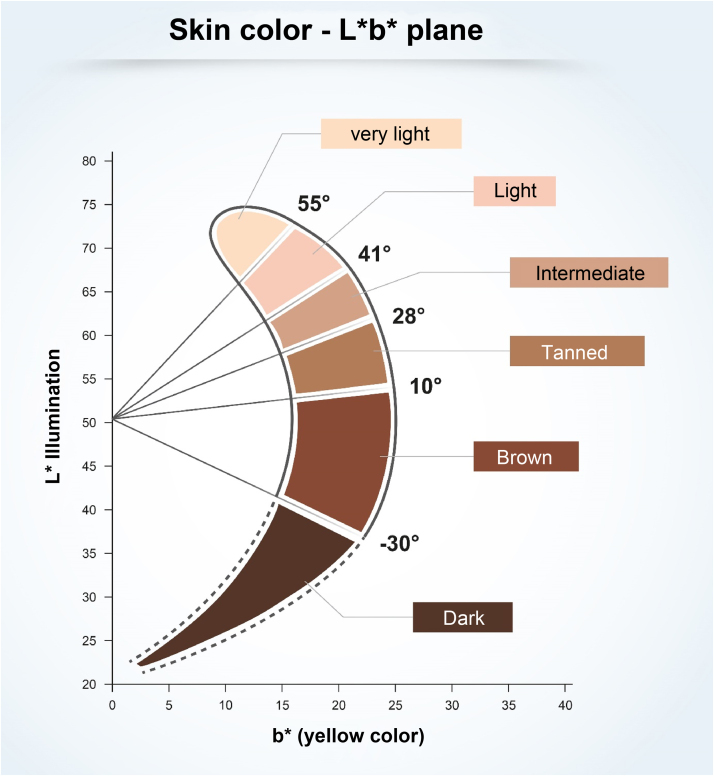
The six skin color categories from very light to dark and their Individual Typological Angle (ITA) values.

To understand the relationship between constitutive skin pigmentation and sensitivity to UV radiation, a study was conducted on 39 *ex vivo* human skin samples, grouped according to Individual Typological Angle (ITA°).^14^ The ITA is obtained through the formula ITA°= [Arc Tangent ((L* - 50) / b*)] × 180 / π, where L* and b*, variables commonly used to measure skin pigmentation, are measured by colorimetry according to the CIELab system (1976), in which a color is identified by three parameters: L* (luminosity, vertical axis ranging from zero (black) to 100 (white); and the horizontal axis a* (color range from green to red) and b* (color range from blue to yellow).^14^ The ITA is inversely proportional to skin pigmentation and allowed the classification of the skin according to its color into six groups, from very light to dark (Fig. 1).^14^ The samples, which ranged from fair to dark skin, were exposed to increasing doses of UV, showing the dose-dependent formation of sunburn cells (UV-radiation-induced apoptotic keratinocytes); they were induced at lower doses in lighter skin.^14^ The analysis of the formed CPDs show that DNA damage was detected in all epidermal layers and in the upper dermis for fair, intermediate, and light brown skin, and only in the suprabasal layers of brown and dark skin, creating a biological relationship with greater susceptibility to photoaging, pigmentation, and development of skin cancer, including melanoma, in lighter skin types.^14^

Recently, it has been shown that visible light and infrared radiation also induce free radicals generated in the skin after exposure to sunlight.^4^, ^15^ Infrared (IR) radiation can transmit energy in the form of heat, raising the temperature of the skin, as well as interacting with other solar wavelengths. It promotes the release of inflammatory mediators in the dermis, which independently induce melanogenesis. IR, UV, and visible light (VL) radiation interact, playing an important role in the development of harmful sunlight effects.^2^, ^15^, ^16^, ^17^

VL and near-IR (type A infrared, at the 750-1400 nm range) can induce pigmentation even in the absence of UV radiation.^2^ Studies suggest that VL emitted by sunlight has a significant photobiological effect on the skin and may induce pigmentation in dark phototypes.^9^, ^16^ It is noteworthy that these photobiological effects of VL are only established with high doses of VL, much higher than those from VL portable electronic gadgets or indoor environments. Recent studies have shown that VL can induce more intense and longer-lasting skin pigmentation than UVA1 radiation, although the effect was enhanced when VL and UVA1 radiation were combined. Moreover, the combination of VL and UVA1 radiation was also able to induce erythema in fair-skinned individuals, an effect previously attributed mainly to UVB and UVA2. Based on these findings, VL and UVA1 may potentially be responsible for aggravating harmful effects of sun exposure, such as phototoxicity in fair-skinned patients and post-inflammatory hyperpigmentation and melasma, especially in dark-skinned individuals.^9^, ^17^

Another study demonstrated that the shorter visible light wavelengths act directly on melanocytes to stimulate melanogenesis and that opsin-3 acts as a blue light sensor on melanocytes, signaling a possible pathway for the prevention of hyperpigmentation by visible light.^16^, ^17^ The main characteristics of visible-light-induced pigmentation in human skin are that it is restricted to melanocompetent individuals (phototype > III) and is long-lasting compared to UVA- or UVB-induced hyperpigmentation.^17^

**Figure 2:**
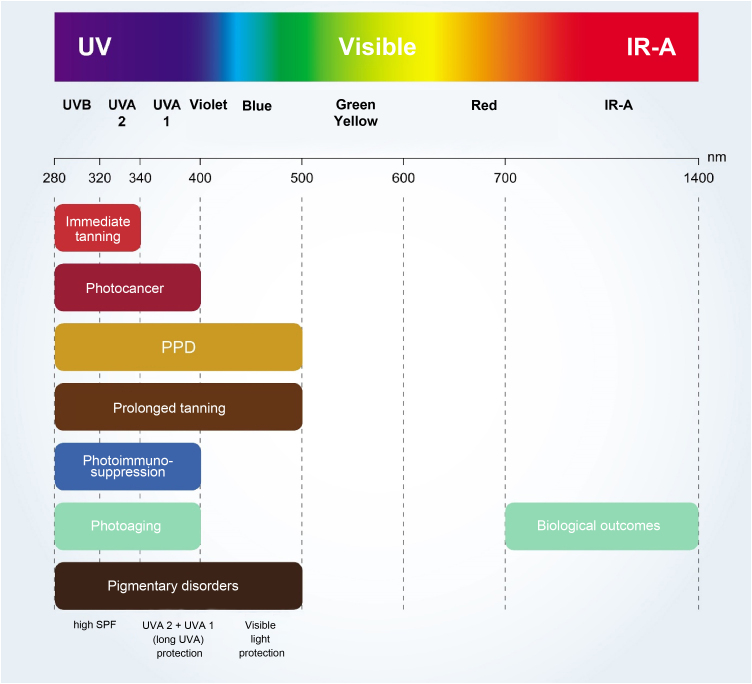
Schematic representation of the effects on the skin of different types of solar radiation. IR-A, infrared - A (780 nm-1.4 μm); PPD, persistent pigmentary darkening.

The critical relationship between the different wavelengths of solar radiation and photocarcinogenesis,^11^ photoimmunosuppression,^2^, ^18^ photoaging,^4^, ^9^ and photodermatosis exacerbation^4^, ^18^ are shown in Fig. 2.

## Photoprotection habits and dermatological practice

In Brazil, according to data obtained from the 21^st^ National Skin Cancer Prevention Campaign of the Brazilian Society of Dermatology (SBD, *Sociedade Brasileira de Dermatologia*) in 2019, it is estimated that 63.05% of people are exposed to the sun without any type of protection.^19^

**Figure 3:**
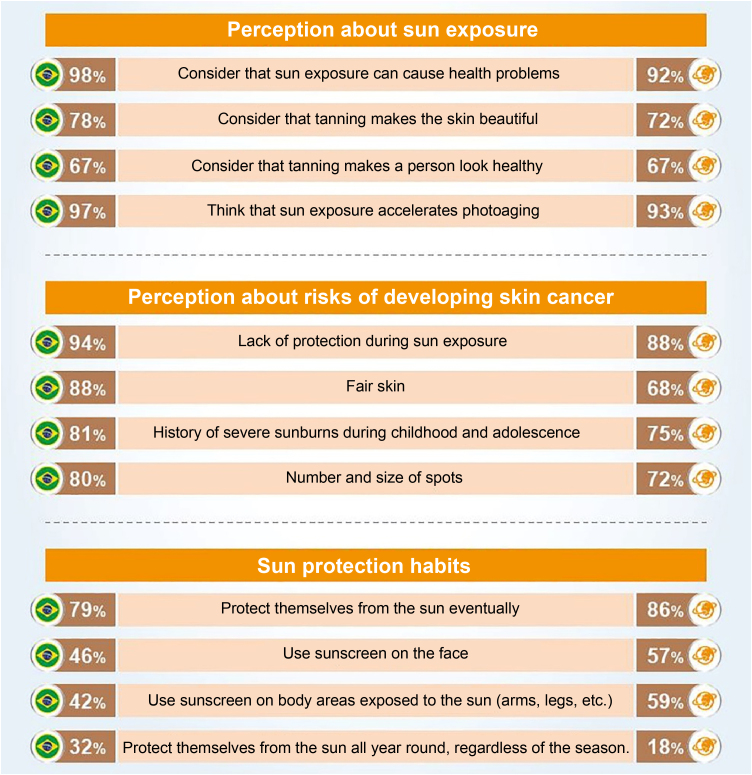
Main parameters of the international research on preventive behaviors regarding sun exposure and photoprotection, comparing the results for Brazil (left column) with the global average among the 23 countries (right column; source: Seite et al.^20^).

An international survey conducted by the Ipsos Institute including 19,569 participants, from Brazil and 22 other countries, aged between 15 and 65 years, on preventive behaviors regarding sun exposure and photoprotection (Fig. 3) places Brazilians in relative alignment with the worldwide opinion, having a perception of sun exposure (98%) higher than the global average (92%) when considering that sun exposure can cause health problems.^20^

Although many are aware of the risks of unprotected exposure, tanning is culturally accepted in Brazil, favoring unprotected overexposure to the sun, especially in childhood and adolescence.^21^ Considering this scenario, a free mobile app (Sunface) was developed and is being used in a cluster-randomized clinical trial with high school students in southeastern Brazil, simulating the effects of unprotected UV exposure on their future faces and evaluating the impact on adherence to UV protection habits, as an educational activity on photoprotection, in a school-related skin cancer prevention program.^21^

A study of 156 American dermatologists was conducted through a live interactive response system survey instrument to determine physicians' perceptions of the safety and efficacy of sunscreens, patient use recommendations, and their personal habits. The dermatologists used several criteria to recommend sunscreen, including SPF level (99%), broad-spectrum protection (96%), cosmetic perception (71%), and photostability (42%). Only 53% of the interviewed dermatologists used sunscreen daily, and 76% generally used sunscreen half the time, with 98.7% of the total recommending the use of sunscreen to friends and family to help protect the skin. When asked about the percentage of patients to whom they recommended sunscreen, 78.8% answered that the recommendation was made to more than 80% of their patients and 99% of the assessed ones believe their patients generally do not apply enough sunscreen.^22^

This perception coincides with several published studies that demonstrate that users apply much less sunscreen than the recommended amount^2^ to ensure uniform skin coverage (2 mg/cm^2^), usually between 0.4 and 1.5 mg/cm^2^.^23^, ^24^

A randomized study evaluated the actual amount of sunscreen (SPF 30 to 45) applied to the face per area (cm^2^) in 101 Brazilian women. The sunscreens used six different vehicles (lotion, mousse, fluid, stick, cream base compact, and powder compact). In a second phase, this study determined the SPF (according to ISO 24444:2010; 2 mg/cm^2^) and the *in vitro* UVA protection factor (UVAPF; according to ISO 24443:2012; 1.3 mg/cm^2^) for the six sunscreens, as well as the SPF and UVAPF (n = 60) based on the actual amounts applied by the individuals in the previous phase, comparing the results. The conclusion of the study was that, in the first phase, the sunscreens were incorrectly self-applied, at lower amounts (0.15 mg/cm^2^ for the powder compact to 1.31 mg/cm^2^ for the lotion) than those recommended by the methodologies, depending on the type of sunscreen used, increasing the risk of DNA changes and showing a deficit in skin protection against UVA and UVB.^24^

## Clinical benefits of daily photoprotection

A study conducted in China with 83 women, aged between 35 and 55 years, evaluated for 6 months the effects of alterations in exposure between winter and summer, through standardized photographs, confirming changes in facial pigmentation. These changes appeared to be reduced or inhibited in the group that was instructed to perform the daily application of sunscreen (SPF 50+/UVAPF 18).^25^

Another study evaluated fourteen clinical signs in facial regions of two cohorts of French women (n = 40 and 42), of similar ages, between 35 and 55 years, during winter and summer. One group was unprotected, while the other applied a sunscreen (SPF 50+, UVAPF 21, critical wavelength = 355 nm) daily, for 6 months, according to the prescribed application guidelines. The blind assessment of clinical signs grouped into four sets: wrinkles and skin texture, ptosis and sagging, pigmentation disorders, and vascular disorders was performed by a panel of experts, based on standardized photographs, and global analysis of the facial pigmentation was determined by colorimetry and spectroradiometry under standardized visible light (CIELab System, 1976), in addition to the determination of the heterogeneity index of pigmentation on the side of the face based on the dispersion of the values ​​of the L*, a*, b* parameters. The unprotected group showed significant changes in summer compared to winter, especially in relation to wrinkles and erythema. These changes induced by the change of season seem to be efficiently reduced in the photoprotected group, which had a more homogeneous, less red and less opaque face color.^26^ The comparison with a previous study carried out in Chinese women, using a similar protocol, shows that the daily use of sunscreen had, in Caucasian women, a more important effect on structural and vascular characteristics than on pigmentation disorders, inversely to the results previously observed in Chinese women. However, both confirmed that the daily application of a broad-spectrum sunscreen decreased facial signs of photoaging, albeit with different impacts according to ethnic or individual-specific characteristics.^25^, ^26^

## Advances in the formulation of sunscreens

Topical sunscreens, defined in the introduction to this article, contain ultraviolet filters (substances or ingredients capable of absorbing, reflecting, or dispersing certain energy from solar radiation), which are combined in a base for the dissemination, homogenization and stabilization of these active substances.^2^, ^7^, ^27^ UV filters must be powerful, offer a broad spectrum of action against UVA and UVB, and maintain unaltered appearance and function when exposed to radiation (photostability). The vehicle must provide a consistent and homogeneous application, remain on the skin without running and leave a pleasant sensation on the skin. Finally, sunscreen must contain a formula that is safe for the individual and that respects the environment.^2^, ^28^

## Photoprotection mechanisms and amplitudes

**Figure 4:**
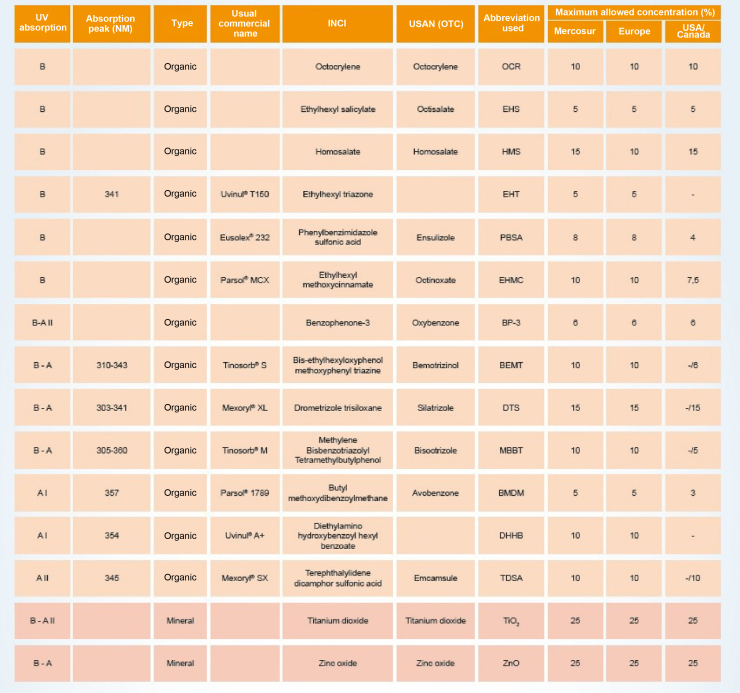
Main UV filters.

The UV filters can be divided into inorganic (physical or mineral) or organic (chemical). Fig. 4 shows a list of UV filters with this classification, which have been approved for use in Brazil (Mercosur),^27^ in the USA and Canada, and in Europe, under their various names, such as the usual commercial name, the American Nomenclature and the International Nomenclature of Cosmetic Ingredients (INCI), whose objective is to technically facilitate the identification of any ingredient not only in Brazil but in any country in the world.^27^

Inorganic filters are particles of mineral origin capable of reflecting or scattering incident UV radiation and/or visible light, acting as a physical barrier. Their main representatives are zinc oxide (ZnO) and titanium dioxide (TiO_2_), with iron oxide pigments also being used. These filters have high photostability, with minimal sensitization potential, and are considered to be broad-spectrum, as they cover the entire ultraviolet spectrum.^2^, ^3^, ^28^ As they do not react with organic filters, inorganic filters are usually used in association with the former. Originally, formulations with physical filters were thicker, not aesthetically pleasing, resulted in an opaque whitish color on the skin transferred to clothing, with consequent impairment of photoprotective efficacy due to the tendency to apply a very thin layer. Since the development of micronized formulations of titanium dioxide and zinc oxide, as well as the encapsulated forms or the ones coated with silica or dimethicone,^28^ inorganic filters have attained greater acceptability, as they enabled the formulation of sunscreens that became transparent after application, although concerns have arisen about the risks of absorption through the skin, not demonstrated by research to date, unless the skin is damaged.^23^, ^28^, ^29^

Organic filters are molecules capable of absorbing UV radiation and transforming it into energetic radiation with a wavelength greater than that of incident radiation, harmless to humans, either in the visible light range or in the infrared radiation range (such as heat).^3^, ^28^, ^30^ The molecule must be able to repeat this process countless times without degrading (photochemical stability). Depending on the specificity of the wavelength range they are able to absorb, organic filters can be divided into UVA filters, UVB filters and, more recently, broad-spectrum filters (UVA and UVB).^2^, ^28^

Generally, UVA filters have their stability compromised when incorporated into the formulation^3^, ^28^ and, depending on their physicochemical characteristics, they are not always able to filter across the entire UVA length range in order to protect the skin from damage caused by UVA2 and, mainly, UVA1 radiation.^13^ Currently, several sunscreens use a combination of UV filters aiming to obtain UVB, UVA2, and UVA1 broad-spectrum protection.^3^ The effectiveness of organic filters is directly related to the photostability of the screening system, vehicle solubility, and water resistance.^28^

To scale the effectiveness of sun protection, the concentration of active ingredients must be adjusted in each formulation, and the combination of organic and inorganic sun filters allows balanced protection through the synergy between the active substances.^31^ Although inorganic filters are broad-spectrum and photostable, the combination with at least one organic filter in the formulation tends to make the texture more pleasant and easier to use,^32^ providing better UVA protection.^3^

### Photostability

Photostability is a critical factor when evaluating the effectiveness of sunscreen in protecting the skin barrier function.^33^ Many molecules lose their UV filtering ability due to photochemical reactions that occur during sun exposure; in addition to leaving the skin unprotected against damage caused by UV radiation, they generate by-products and intermediate compounds of these reactions, which are reactive oxygen species (ROS), or other cytotoxic molecules that can sensitize and further damage the skin. Photoinstability is common even in commercially available sunscreens and therefore, the need for standardized and robust photostability measurements is highlighted.^32^, ^33^, ^34^

For many years, butyl methoxydibenzoylmethane (BMDM) was the only organic filter to provide protection against UVA1 radiation allowed in Europe and the US. However, this active substance undergoes significant degradation when exposed to light and is often combined with UVB filters (Octocrylene, OCR; Benzophenone-3, BP-3) and inorganic filters to become photostable and extend the coverage spectrum.^3^, ^28^, ^34^

Due to the high relevance of photostability for the sunscreen to be effective, several strategies^28^ have been used: combination of UV filters that stabilize each other against photodegradation^31^; micro and nanoencapsulation of UV filters^2^, ^32^; addition of antioxidants, such as vitamin E^3^, ^17^ or caffeine^35^; and development of structurally photostable molecules, such as the cis/trans isomerization phenomenon that occurs in terephthalylidene dicamphor sulfonic acid (TDSA).^18^

Few inputs are available that cover photostability and protection efficacy criteria at wavelengths greater than 340 nm, where the skin permeation of UV radiation is maximized, with TDSA standing out, which has a well-balanced, photostable molecular structure, with a peak of absorption at 345 nm, at the limit between UVA2 and UVA1 wavelengths.^18^ As it is hydrophilic, it also has synergistic properties when combined with lipophilic organic filters. When it is combined with BMDM, there is an increase in UVA protection. *In vivo* studies have shown protection against photoaging and the development of photodermatoses.^18^, ^28^ Pyrimidine dimer formation and p53 accumulation were significantly reduced by TDSA formulations, leading to greater efficacy against a large number of biological lesions induced by sun exposure.^18^

Another more recently introduced organic filter is drometrizole trisiloxane (DTS), not yet approved by the FDA. DTS absorbs both UVB and UVA2 radiation and is highly capable of UVA protection when combined with TDSA.^21^, ^28^

A comparative study of fifteen SPF 20 sunscreens marketed in Europe evaluated the photostability of each product through the area under the absorption curve (AUC) for the total UV range and for each radiation range separately (UVB, UVA, UVA2, UVA1); and the residual efficacy of SPF and UVAPF, both *in vitro*, measured by changes in the absorbance profiles of sunscreens before and after exposure to sunlight.^34^ All fifteen sunscreens were photostable in the UVB region; seven products, all containing a combination of ethylhexyl methoxycinnamate (EHMC) and BMDM with other UV filters, exhibited photoinstability in the full UV spectrum (290–400 nm), justifying the importance of stabilization strategies for BMDM; and eight products lacked stability in the UVA1 range, confirming the difficulty of organic filters in not photodegrading in this wavelength range. Only five sunscreens showed excellent or very good UVA1 photostability (AUC-UVA1 index above 0.93), being mentioned in the publication as S8 (EHMC, ethylhexyl triazone (EHT) e methylene bis-benzotriazolyl tetramethyl butylphenol (MBBT); AUC-UVA1 of 1.00), S10 (TiO_2_ and ZnO; AUC-UVA1 index of 0.98), S4 (EHMC and bis-ethylhexyloxyphenol methoxyphenyl triazine (BEMT); AUC-UVA1 index of 0.97), S12 (EHMC, MBBT and TiO_2_; AUC-UVA1 index of 0.96) and S15 (OCR, EHT, BMDM, TDSA, DTS and TiO_2_; AUC-UVA1 index of 0.93).^34^ This result highlights the importance of photostability assessment to ensure the effectiveness of sunscreen^32^, ^34^; however, this is not usual information on the product packaging, nor is it a regulatory requirement in Brazil or in other countries.^3^, ^7^

### Antioxidants

In addition to possibly aiding in the photostabilization of UV filters, topical antioxidants reduce the production of ROS, cytokines, and type 1 matrix metalloproteinase expression following UV and visible light irradiation.^9^ Thus, they are not considered sunscreens, but they are interesting additives to stabilize and attenuate the oxidative effect of UV radiation and even pollution, protecting the skin from oxidative damage and helping to reduce the risk of non-melanoma skin cancer.^1^, ^9^, ^36^

Antioxidants at the concentrations normally used in sunscreens did not influence the SPF value, nor did they delay the erythematous response, according to an *in vivo* study that compared the protective efficacy against UVB radiation (ISO 24444:2010) of an SPF 30 sunscreen (control) with SPF 30 sunscreen formulations containing several antioxidant/active anti-inflammatory substances, such as vitamin E, glycyrrhetinic acid, panthenol or *Glycyrrhiza inflata* extract, although the effects of antioxidants on skin alterations resulting from sub-erythematous exposure to UV radiation have not been evaluated, nor the responses to other radiations (UVA, VL and IR).^36^ While sunscreens are designed to act on the skin surface as protective shields, antioxidants are designed to work both on the surface and in the layers of viable skin cells. The use of a topical antioxidant product as an adjuvant is expected to be a logical complement to sunscreens, particularly as they do not need to physically remain on the skin surface during sun exposure, as required for sunscreens.^1^, ^2^, ^9^

### DNA repairers

The inclusion of antioxidants and DNA repair enzymes in topical sunscreens has extended the approach for an active photoprotection, capable of enhancing the protective power of traditional sunscreens.^9^, ^28^ Photolyases are enzymes that occur naturally in bacteria, plants, and animals that experience high exposure to UV rays.^9^ In the presence of flavonoids that absorb UV radiation and transfer excited electrons to CPDs, photolyase acts to repair DNA.^9^ Patients being treated with photodynamic therapy for actinic keratoses (AK) who used sunscreen containing topical photolyase had longer remission times.^9^

Although endogenous DNA repair mechanisms are relatively effective, some damage persists and can accumulate with chronic exposure. Increased DNA repair effectiveness and genomic stability of skin cells have been observed with nicotinamide, also called niacinamide.^37^ Nicotinamide is a precursor of nicotinamide adenine dinucleotide (NAD+), an essential coenzyme to increase the production of adenosine triphosphate (ATP) that prevents the cell energy crisis induced by UV radiation. This action not only enhances DNA repair but also reduces UVA1- and UVB-induced immunosuppression and photocarcinogenesis,^37^, ^38^ and p53 gene expression has been shown to be up-regulated by nicotinamide in the presence of UV.^38^ One study showed that nicotinamide reduced CPDs and 8-oxo-2-deoxyguanosine (8oxoG) in an *in vitro* model with human keratinocytes and in an *ex vivo* human skin model, in two possible pathways for repair of UV-induced lesions.^37^ A randomized, double-blind, placebo-controlled pilot study was carried out in elderly patients to assess the effect of topical 1% nicotinamide on AK compared to vehicle, both applied twice a day. The patients treated with nicotinamide had a significant 22% reduction in AKs at 3 months, compared to a non-significant reduction with a vehicle, showing a faster rate of resolution of AK with nicotinamide than with vehicle in a group of individuals with skin that was very badly damaged by the sun.^39^ This and other studies support the potential of nicotinamide as an active substance that may be effective in preventing and treating actinic keratosis.^37^, ^38^, ^39^

### Vehicles and cosmetic texture

The development of an appropriate vehicle for sunscreen is of utmost importance, as viscosity and emulsifying agents influence the stability, sensory properties and surface tension of sunscreens, modulating the distribution of UV filters and the formation of a uniform film of the product on the skin.^40^ Currently, sunscreens are available in the most varied forms, adapted to the climate and habits of each country, such as fluid, cream, gel and aerogel, lotion, mousse, spray, serums, dry-touch texture, among others, and the type of vehicle and application conditions affect film thickness, which ultimately influences the effectiveness of the SPF.^40^ Additionally, although protection effectiveness is a requirement, clinical practice demonstrates that textures with a more pleasant touch and spreadability have greater cosmetic acceptability and increase patient compliance with the use of sunscreens.^2^, ^41^

**Figure 5:**
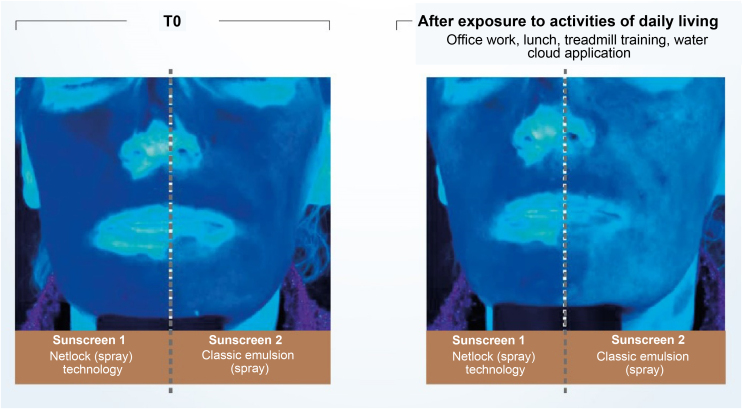
Illustration showing UV radiation absorption capacity of two sunscreens evaluated by photographs taken under UV light. (Source: Moyal et al.^31^).

Recent publications reinforce the importance of uniform application in adequate quantity and periodic reapplication (every 2 hours).^2^, ^4^, ^30^ No uncovered skin should be left without sunscreen protection when applying the product, and all exposed areas should be included.^30^ The ideal film on the skin must have continuity, constant thickness, and a fine and homogeneous distribution of UV filters, which is made difficult by the fact that the skin is not smooth and flat.^30^ The thicker the layer of sunscreen applied to the skin, the more regular film tends to be formed and the higher the SPF and UVAPF.^3^, ^40^ Another important aspect affecting sunscreen effectiveness is the vehicle capacity to form a coherent and consistent enough film to prevent migration of the formula over the skin.^3^, ^28^ Technologies based on semi-crystalline polymers, for example, stabilize the filters in the formulation without the aid of surfactants, resulting in a film with constant thickness, uniform coverage, and an even distribution of filters on the skin. Studies conducted with this technology (INCI C12-22 alkyl acrylate/hydroxyethylacrylate copolymer, L’Oréal, Clichy, France – NetLock Technology) demonstrated its ability to increase the sun protection factor and promote film resistance after performing daily activities. In this study, the UV absorption of two sunscreens was evaluated through photos taken under exposure to UV light (Fig. 5). After activities such as office work, treadmill running, going to lunch, and showering, the UV absorption of the classic emulsion was noticeably reduced, while the emulsion with the technology based on semi-crystalline polymers remained unchanged.^31^

Some vehicles, mainly because of the amount in which they tend to be applied, require special advice from dermatologists.^4^, ^22^ Sunscreens in cosmetic powders should not replace traditional topical sunscreens due to their lower protective efficacy, but they are great options to reinforce UV protection; improve the visual appearance, reduce skin shine, produce a uniform skin color and texture; or on reapplication throughout the day for maintining the SPF.^2^, ^24^ Spray sunscreens have shown to be as effective as lotions when applied correctly,^4^ with increasing user acceptance, albeit with questionable practical results due to the difficulty to adequately cover all exposed parts, distribute the product evenly and use the recommended amount.^2^ In the US, the preference has shifted from lotions to sunscreens in clear sprays, mainly because of the quick and easy application of these products.^42^ The lack of specific guidelines for the use of spray sunscreens and concerns about safety and adequate application by the patient may explain the hesitation of some American dermatologists to recommend sprays, as seen in a recent survey, where 99% of 540 dermatologists recommended sunscreens to their patients, but only 69% recommended the spray formulations.^42^ At the time of reapplication, spray formulations can be helpful in enhancing the effect of another sunscreen.^2^

In Brazil, due to the frequent demand for sunscreens that reduce skin oiliness, there is a large number of oil-free preparations, or those with components that adsorb lipids from the skin, such as different silica structures, comprising a product line with controlled skin oiliness, even suitable for the treatment of adolescents with acne.

Recommendations on the prescription and use of a higher SPF have been considered a strategy, from the scientific point of view, to attain more effective sun protection and compensate for the inappropriate application of sunscreens.^4^, ^23^ The evolution in sunscreen development regarding cosmetics^4^, ^28^ and the current offer of textures adapted to different skin types made it possible to use a higher SPF, which reinforces this recommendation. Special attention, including a targeted approach, should be given when choosing the vehicle for specific populations, such as sportspeople who practice outdoor physical activities and outdoor workers.^2^, ^23^

### Individual safety of sunscreens

UV filters are intended to act on the surface of the skin, protecting it from the harmful effects of sun exposure, and systemic absorption could undermine its assessment of efficacy and safety.^43^ However, the stratum corneum seems to be an effective barrier against penetration of UV filters, and the risk of significant systemic absorption should not be a concern. The most recent UV filters, most not yet available in the US, tend to have a high molecular weight and, regardless of the formulation, do not penetrate the skin, increasing the level of safety in the formulations.^23^, ^29^

Currently, there is a growing public concern about the harmful effects of chemicals in sunscreens.^3^, ^23^ The most common adverse effect of using sunscreens is the risk of photoallergy, particularly with benzophenone class organic UV filters, which are considered allergenic UV filters.^28^, ^44^, ^45^

The literature on BP-3, the main representative of the benzophenone class, indicates that it is absorbed by the skin to a greater extent than previously understood and can lead to significant systemic availability, in addition to data showing the presence of BP-3 in human breast milk, amniotic fluid, urine, and blood plasma and some studies that have shown estrogenic and anti-androgenic effects; however, the clinical relevance of these findings remains uncertain. 4, 9, 23, 45, 46, 47

In Europe, BP-3 has been largely replaced by other broad-spectrum UV filters, but this replacement cannot easily be done in the US because many of these filters have not yet been approved by the FDA.^9^ There are also rare occurrences, regarding the widespread use in sunscreens, of contact dermatitis and photoallergy, particularly with cinnamate derivatives such as EHMC and OCR.^23^

Based on the current safety data available, OCR used as a UV filter in cosmetic products at a concentration of 10% can be considered safe, as there has been no evidence of potential endocrinological imbalance, as experimental studies have shown no adverse effects on reproduction and development parameters; and thyroid effects reported in repeated dose toxicity studies performed in an animal model at very high doses are species-specific and not relevant considering the doses of octocrylene used in humans.^48^ Nevertheless, the concern of formulators in achieving the highest UVA protection with the lowest possible total amount of organic filters to avoid adverse reactions, such as skin irritations and allergies; potential environmental impact; and ensure acceptable texture for better application and use of sunscreens is still valid.^4^, ^9^, ^41^

Sunscreen developers must select not only safe active substances but also preservatives, surfactants, emollients, emulsifiers, and fragrances to compose an excipient or vehicle with the least possible potential to sensitize the skin. In this sense, a survey of allergens in 910 dermatological products available in the Brazilian market indicated fragrance (62.2%) as the most frequent one, representing 73.3% in the category of analyzed sunscreens (n​​ = 145).^49^

On the other hand, mineral UV filters are rarely allergenic^2^ and, although controversy has recently arisen regarding the systemic absorption of micronized particles from these inorganic filters, studies have not shown potential damage and reinforce the role of these active substances in protecting the skin against adverse effects induced by UV radiation, including DNA damage and skin cancer.^3^, ^23^ A review article has gathered information that reinforces that titanium dioxide nanoparticles (nano-TiO_2_) are considered non-sensitizing and mildly or non-skin irritating.^29^ Furthermore, there is no evidence of carcinogenicity, mutagenicity or toxicity following dermal exposure to nano-TiO_2_; no cytotoxic effects were reported using a 3D human skin model; and nano-TiO_2_ does not penetrate the skin or reach the circulation after being applied to healthy or compromised skin, showing no health risks in concentrations of up to 25%.^29^ In Europe, the only restriction on the use of nano-TiO_2_ is in formulations that can lead to lung exposure through inhalation, i.e., spray and powder products.^29^

Concerns about the adverse effects of sunscreens stimulate dermatological research and investigations. Observational studies have reported a positive correlation between facial sunscreen use and risk of frontal fibrosing alopecia (FFA), but the review of the available literature did not provide sufficient evidence to establish a direct causal relationship between sunscreen and FFA.^50^ In a recent study conducted in Brazil with 451 women diagnosed with FFA, compared to women without FFA, no finding associated the use of sunscreen to FFA, although a possible relationship of FFA and hair straightening with formalin or even with nighttime facial moisturizer has been identified.^51^

To date, there is not enough data to support the fact that sunscreens cause toxicity in humans, but there are signs that indicate that further studies are necessary, particularly on the effect of certain UV filters on the environment.^4^, ^9^, ^45^, ^52^

### Environmental safety of sunscreens

UV filters have been reported as a threat to coral reef coastal ecosystems.^9^, ^47^ In real-world conditions, when assessing all threats to corals, sunscreen does not seem to be the main issue. Global climate changes, pollution and sewage are some of the main factors contributing to coral degradation.^47^, ^53^

The symbiosis relationship between corals (hosts) and single-celled algae (dinoflagellates) is currently the object of much debate due to extensive records of bleaching of the world major reefs.^45^, ^53^ Bleaching corresponds to the breakdown of coral-dinoflagellate symbiosis, the subsequent loss of symbionts that provided their hosts with photosynthetic products for their energy needs and gave them the characteristic color. When coral becomes stressed, it sheds algae, becomes whitish, and starves due to the loss of its main food source.^52^, ^53^

To verify the photochemical response of a coral to sunscreen ingredients, a study evaluated the chronic effects of organic (TDSA, DTS, EHT, BMDM, and OCR) and mineral (ZnO) UV filters on the photosynthetic efficiency of symbionts associated with the coral *Stylophora pistillata*. The results indicated that TDSA, DTS and OCR, at relevant concentrations (higher than those reported in seawater) and taken individually for 5 weeks, did not induce bleaching, nor did they cause a significant decrease in the photosynthetic efficiency of the corals. EHT and BMDM showed a decrease in the concentration, suggesting that these products were adsorbed on the fish tank glass or were degraded by photoexposure.^53^

On the other hand, ZnO, which is classified as “hazardous to the aquatic environment” according to the Globally Harmonized System of Classification and Labeling of Chemicals (GHS) criteria, appeared as the most toxic compound. Other studies have shown that the marine impact of dissolved and nanoparticulate forms of ZnO should be carefully evaluated on endangered coral reefs.^47^, ^53^

TiO_2_ caused minimal changes in symbiotic relationships and did not cause bleaching in corals of the *Acropora* genus, resulting in greater eco-compatibility.^47^ BP-3, found at detectable levels from 0.8 mcg/L to 1.4 mg/L in seawater, has been shown to cause coral bleaching *in vitro*,^47^, ^52^ while another study evaluated the exposure of fish belonging to the species *Poecilia reticulata* to this filter, and the results showed that BP-3, at an environmentally relevant concentration, was genotoxic to freshwater fish, corroborating its environmental risk.^54^

### Sunscreens and hypovitaminosis D

The benefits of solar radiation are mainly related to vitamin D synthesis and the prevention of diseases such as osteoporosis, type 1 diabetes, cancer of the colon, breast, prostate, non-Hodgkin lymphoma, and autoimmune diseases.^5^ Most of the vitamin D is obtained by cutaneous synthesis through sun exposure, and the wavelength of solar energy that causes erythema considerably overlaps in the ultraviolet B region with that which synthesizes vitamin D in the skin. Theoretically, sunscreens that inhibit erythema should also inhibit vitamin D synthesis, which would be a possible adverse effect of sunscreen use, with public health implications. However, in practice, sunscreens can be used to prevent sunburns and still allow vitamin D synthesis.^6^, ^55^

A quasi-experimental, randomized study carried out in the city of Rio de Janeiro (Brazil), during the winter, involving 95 healthy adults, evaluated the vitamin D synthesis induced by suberythematogenous sun exposure of a group under topical photoprotection (SPF 30), a group exposed to the sun without sunscreen and a group confined from sun exposure that received doses of 25-OH-vitamin D, before and 24 h after sun exposure (UVB = 20 mJ/cm^2^). Even after applying sunscreen on the face, neck and thorax, sun exposure induced a variation in vitamin D levels, comparable to that obtained without photoprotection.^55^ The use of sunscreen for daily and recreational photoprotection does not compromise vitamin D synthesis, even when applied under ideal conditions.^56^

One article presented the UV Index (UVI) measurements carried out in São Paulo (Brazil), in the period of 2005–2008, when 65% of the UVI measured 2 hours around the local midday during the summer showed very high levels (8 < UVI < 10) and extreme levels (UVI > 11), according to the WHO classification. During the winter, 40% of measurements around midday showed high or very high levels. UV measurements have also shown that in every month of the year, UV radiation levels are high enough to ensure vitamin D synthesis in the skin with minimal sun exposure for 10 minutes^57^; another study suggested sun exposure at noon twice a week for 5–30 minutes as sufficient for vitamin D production in white populations.^2^

Recently, the WHO recognized that sensible sun exposure offers health benefits, including vitamin D production.^8^ However, conscious exposure with the use of an efficient sunscreen is recommended.^2^ Regarding patients over 60 years of age, the suspension of daily use of sunscreen does not help in the synthesis of vitamin D, since it has been shown that the ability to produce vitamin D3 from the same UV exposure decreases with age,^58^ and can be reduced to about 50% of a child's capacity to fully synthesize vitamin D in the skin for these patients.^58^ Even with conscious and well-guided sun exposure, oral supplementation is indicated for patients at risk of developing hypovitaminosis D.^2^

## Stringency in determining the effectiveness of sunscreens

The methods and standardizations of sunscreen performance evaluations aim to guarantee the reliability of the products in search of the safest and most reproducible topical photoprotection. The International Organization for Standardization (ISO) and the FDA are working on global harmonization of methods for assessing UV protection, focusing on SPF and UVA, both *in vivo* and *in vitro*.^3^

### Regulation of sunscreens

The regulations for sunscreens vary between countries and, in a globalized world where patients have access to sunscreens from all over the world, and it is important that dermatologists know the main regulatory differences.

In Brazil, the National Health Surveillance Agency (Anvisa, *Agência Nacional de Vigilância Sani*tária) defines filters or sunscreens as cosmetic products in accordance with RDC N. 30 of June 1, 2012,^7^ which approves the Mercosur Technical Regulation on Sunscreens in Cosmetics and has a list of permitted UV filters containing 39 active ingredients in RDC N. 69 of March 23, 2016.^27^ Even though they aren’t medications, sunscreens must follow the technical requirements, labeling criteria, and efficacy assessment methods defined in the legislation to ensure its effectiveness and a high level of protection for the individual.^7^, ^27^

North-American legislation classifies sunscreens as over-the-counter (OTC) drugs. In the US, only 16 UV filters have been approved by the FDA in the sunscreen monograph; an additional filter, ecamsule (TDSA), has been approved through a different process (New Drug Application) and has been used in US formulations. A new UV filter has not been approved in the US for over 10 years, leaving US manufacturers limited to offering broad-spectrum UV protection comparable to sunscreens from other parts of the world.^4^

In 2019, the FDA proposed new rules on sunscreens, and a series of preliminary studies were spurred to support data on the safety and efficacy of UV filters aiming to support the new classification of filtering actives into “generally recognized as safe and effective”.^47^ Consequently, studies on the risks of systemic absorption and detection of plasma concentrations of UV filters have been published, which do not imply toxicity or that they are harmful to health.^59^ In addition, the FDA has proposed increasing the maximum SPF value reported by manufacturers from 50+ to 60+ and adding a test to ensure broad and uniform protection against both UVB and UVA.^60^

In Europe, sunscreens are classified as cosmetics; however, quality requirements are equally high; the allowed UV filters are regulated by Annex VI of the Cosmetics Regulation (EC N. 1223/2009), and the parameters established for labeling are: SPF with a maximum value of 50+ and UVAPF with a value of at least one-third of the UVB protection.^3^, ^61^

The importance of adequate protection from UVA radiation has increased and the United States and Europe have different regulatory standards for UVA protection in sunscreens. One study evaluated 20 US sunscreens (broad-spectrum; SPF 15 – 100+); nineteen of the 20 products (95%) had a critical wavelength >370 nm, an FDA requirement for registration of UVA protection. However, only eleven of the 20 (55%) products would meet the European standard for the definition of “UVA protection”, which is to have a UVAPF to SPF ratio of at least 1/3.^62^

### Sun Protection Factor (SPF)

The sun protection factor (SPF) is an international reference for expressing protection against UVB radiation,^3^ being in practice a primary measure of protection against UVB radiation and, to a lesser degree, against UVA2 radiation. Currently, sunscreens are manufactured in a wide range of very high sun protection factors, with SPF < 99 being allowed in Brazil. In parallel, the concern with the UVA protection of sunscreens has gained importance and the performance of each sunscreen in relation to UVB and UVA protection must be understood both in terms of quantity (level) and quality of protection.^3^, ^60^

In Brazil, two methodologies are accepted for determining the SPF: ISO 24.444:2019 (also used in Europe, Canada, Australia and Japan) and FDA 1999, 2011 (used in the USA), both performed *in vivo*, and with the evaluation of the erythematous reaction, on the dorsal region, after application of 2.0 mg/cm^2^ of sunscreen. The SPF is the value obtained by the ratio between the minimal erythematous dose (MED) on the skin protected by a sunscreen and the MED on the unprotected skin, with MED being the minimum amount of ultraviolet radiation required to produce the first evident erythematous reaction, covering more than 50% of the exposure subsite and with clearly defined edges, observed between 16 and 24 hours after exposure to UV radiation, according to the adopted methodology,^7^ and, therefore, it is a measure of protection time in the laboratory, and not of sun protection intensity.

Although the assessment of SPF is carried out using standardized and established methods (ISO or FDA methodologies), it is possible to find variability in the results. This variability, inherent to the methodologies, demands a process with high-quality requirements to ensure that the result is reliable, reproducible and that guarantees protection to the consumer or patient. The criteria that impact the SPF oscillation include inter-individual variation, product application method, UV exposure, and the definition and reading of the minimal erythematous dose. Given the vulnerabilities of the standardized methodology, some companies develop quality policies, internally and externally, together with pre-qualified partner laboratories, for highly collaborative work, aiming to obtain a more authentic and reliable SPF and stimulate research and discussions on the evolution of methods to assess photoprotection with the ISO group and photoprotection experts.^63^

Given the complexity of measuring the SPF of a sunscreen, the formulation of individual sunscreens in compounding pharmacies practically makes it impossible to adequately determine the SPF, leading to an empirical establishment of the SPF value, without guaranteeing the photostability of the filtering system, since it is unfeasible to determine, in a robust way and with quality, the level of performance of the small quantities produced in different master formulations and vehicles.

According to the scientific community that studies the topic, the SPF is the only validated methodology to assess the effectiveness of sunscreens, as it correlates with the clinical result (erythema) of protected and unprotected skin. However, the SPF is not linear, and the same SPF does not mean that different sunscreens have the same biological effects.^64^ A mistake is to relate SPF to exposure time, which is only possible in the laboratory since the test conditions (amount of product, amount of radiation, etc.) are controlled. The erythematous response is directly related to the type of skin and, in real-life conditions, it depends on the amount of radiation, which can vary with the place and time of sun exposure and with the amount of product applied to the skin. SPF values could also be understood ​​as multiplicative ones, making one draw the conclusion that SPF 30 is twice as effective as SPF 15, for instance. However, an SPF 15 sunscreen absorbs 93.3% of the radiation that induces erythema; an SPF 30 sunscreen absorbs 96.7%; while an SPF 50 sunscreen absorbs about 98% of UVB radiation. It is more clinically and photobiologically relevant to measure the amount of electromagnetic radiation being transmitted to the skin, whether 6.7% for SPF 15; 3.3% for SPF 30 and 2% for SPF 50 (which means increasing the protection by more than three-fold when compared to SPF 15).^2^, ^4^ In Brazil, where the radiation level is very high, the use of sunscreen with a higher SPF is important to reduce the amount of radiation that is passing through the filter and reaching the skin.^9^, ^23^

### UVA Protection Factor (UVAPF) and Critical Wavelength

**Figure 6:**
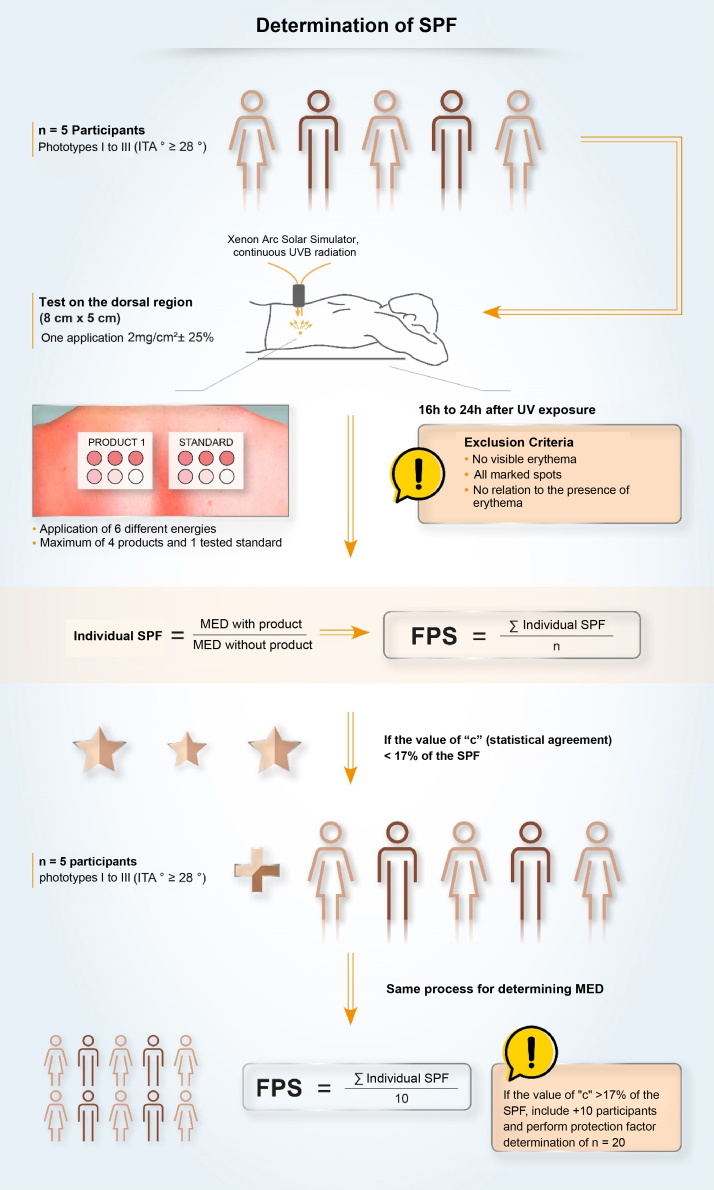
Sun protection factor (SPF) evaluation method.

Once the importance of broad protection (UVB and UVA) was understood, it was necessary to harmonize and standardize the measurement conditions aiming to transmit adequate information about UVA protection to the consumers. The studies by Moyal et al. (L'Oréal Recherche, France) demonstrated the relevance of persistent pigment darkening (PPD) as a biological outcome to assess UVA photoprotection, as well as the sensitivity and reproducibility of the *in vivo* methodology for the variety of UVA filters^65^ and, later, Moyal participated, together with COLIPA, in the development of a new *in vitro* method to measure UVA protection,^66^, ^67^ which is correlated with the *in vivo* measurement. In the *in vivo* method, the UVA protection factor (UVAPF) is the value obtained by the ratio between the minimal pigmentary dose (MPD) on a skin protected by a sunscreen and the MPD on the same skin, when unprotected, with the MPD being the minimal dose of UVA radiation required to produce a persistent pigmentary darkening of the skin with clearly defined edges, observed between 2 and 4 hours after exposure to UVA radiation.^7^ In Brazil, a product can only be registered as a sunscreen (Fig. 6) if the protection against UVA radiation is at least one-third of the protection against UVB radiation (SPF) and the critical wavelength is at least 370 nm, to ensure protection against long UVA.^4^, ^7^ The accepted methodologies for the determination of UVAPF are ISO 24442:2011 (*in vivo*) and ISO 24443:2012 (*in vitro*) and the results can be correlated.^7^ The ISO 24442:2011 methodology to establish UVA protection is similar to the ISO 24444:2010 used for the SPF, regarding the number of homogeneously selected volunteers, except that they are phototypes III to IV (Fitzpatrick), which will be irradiated to observe skin pigmentation.

**Figure 7:**
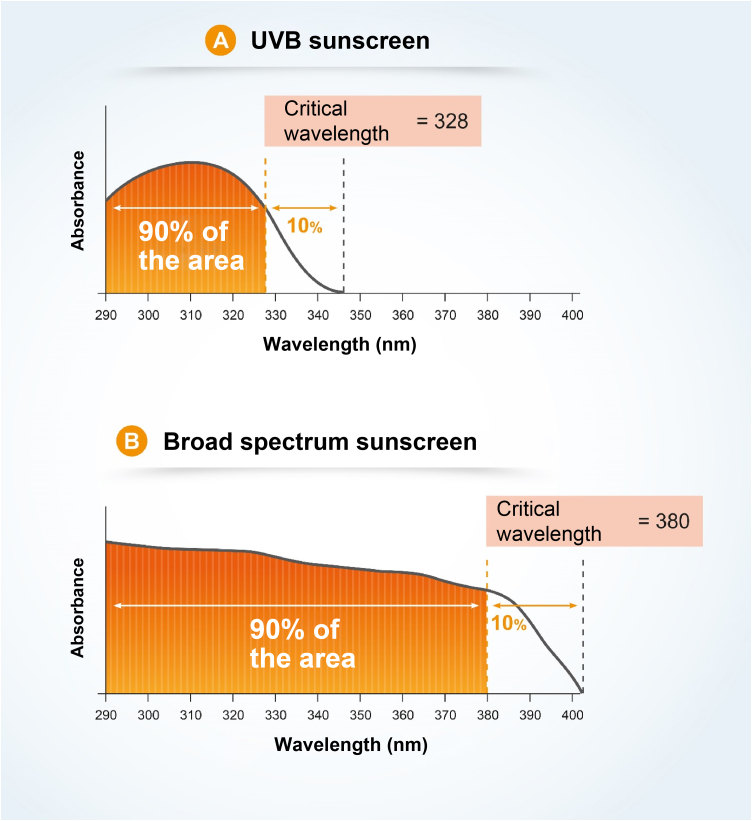
Critical wavelength is a quality characteristic and allows for a statement about the scope of UV protection. **A**, Product with high UVB absorption and low UVA absorption. **B**, Product with uniform UVB and UVA absorption.

The critical wavelength (λc) is defined as the one for which the area under the integrated optical density curve, starting at 290 nanometers, is equal to 90% of the integrated area between 290 and 400 nm (Fig. 7).^7^, ^13^

### Visible light (VL) and infrared radiation (IR)

The development of sunscreens with good UVB and UVA efficacy favors safer sun exposure; however, when there is no protection in the VL and IR spectral region, large amounts of free radicals are induced.^15^ The effectiveness of sunscreens can be extended to also cover the visible and infrared spectral region of solar radiation, adding pigments and antioxidants to the formulations capable of fighting the free radicals that are formed.^4^, ^15^ Several validated methodologies are being presented in the literature, but there is no universally accepted standard as yet.^68^, ^69^

A cross-sectional study was carried out to evaluate the *in vitro* transmittance to UVB, UVA and blue-violet light (400–500 nm) of 41 Brazilian commercial sunscreens, with SPF > 30, all opaque; and three more pigment-free sunscreens (control). The opaque filters had greater coverage than the controls, but only 63% (26/41) blocked >99.9% of VL, while some sunscreens that had high protection against VL let UVA radiation through, contradicting the hypothesis that all pigmented sunscreens protected from VL and UVA radiation.^70^

A VL assessment protocol to verify the capacity of a formula to prevent VL-induced hyperpigmentation showed that there is a correlation between the amount of iron oxide and the effectiveness of protection against visible light (VL); however, there are different studies and diverse parameters that demonstrate that it is possible to obtain good efficacy even with a reduction in the concentration of iron oxide. An iron oxide-free, titanium dioxide-only sunscreen offered 14% greater protection against VL than the unprotected skin in terms of reduced ITA value after 5 days.^17^, ^68^

### Assessment of water-resistance capacity

In Brazil, water resistance is regulated and must strictly follow the same methodology selected to determine the SPF (FDA or Colipa). In the US, the FDA prioritizes immersion in water and only then evaluates the SPF; while in the European guidelines (Colipa), water-resistance can only be claimed if more than 50% of the SPF is retained after the water immersion procedure: two 20-minute immersions for water-resistant products or after four 20-minute baths for very water-resistant products.^3^, ^7^

### Photostability check

The current performance measurements, SPF *in vivo* and UVA *in vitro* ISO 24443, conducted under standardized conditions, are partly responsible for the photostability of the final product.^34^ Photostability is not a regulatory requirement but can be assessed by comparing the *in vitro* absorbance of sunscreen before and after exposure to UV radiation.^32^, ^33^

### Sunscreen labeling

**Figure 8:**
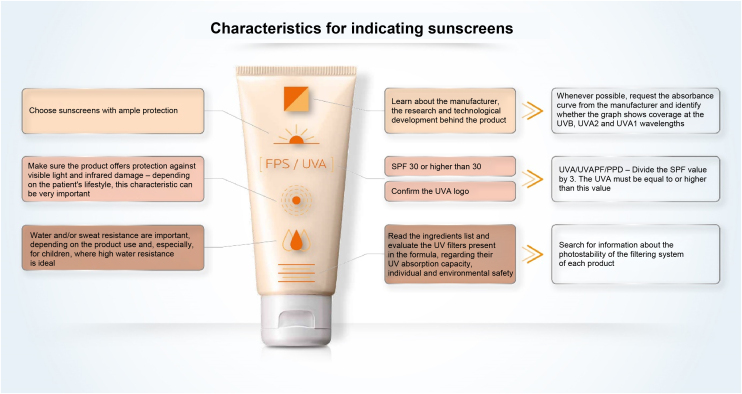
Important characteristics for sunscreen indication.

When looking at the packaging of sunscreen for dermatological use, the comparison between products tends to be impossible for the health professional and is even more misleading for the common consumer. The SPF is the information most commonly related to the effectiveness of sunscreens and the easiest to see on the sunscreen label (Fig. 8). However, its interpretation should not be based only on the numerical value itself, as it should also consider the adequate way to use the product in terms of applied quantity and regularity of reapplications. In Brazil, the SPF value can vary from 6 to 99 and, according to the pre-established category, sunscreens with an SPF between 30 and 50 are labeled as “High Protection” and products with an SPF between 50 and 99 are labeled as “Very High Protection”. These phrases refer to UVB protection only.

UVAPF can be represented on the packaging by a logo (); simply by mentioning “UVA”; describing UVA protection factor; or even PPD, a method used to measure protection against UVA radiation.

The ingredients are presented in all sunscreens, listed by the INCI nomenclature, in descending order of concentration in the formula, without highlighting each category of active substances or UV filters; that is, one needs to know the names and locate them in the list of ingredients.

The information on the label or packaging tends not to inform about the absorption peaks of the filtering system nor about its photostability.

A study to evaluate the effectiveness of protection against VL and IR radiation has not yet been agreed on, but by presenting studies in validated protocols, the product may exhibit the information “protects against visible light” and “protects against damage caused by infrared radiation”.

Finally, when choosing a sunscreen, it is also necessary to verify the information regarding water and/or perspiration resistance according to the consumer habits.

Cosmetic acceptability, particularly touch and visual appearance, should also be considered for better adherence to sunscreen use.^30^

## Final considerations

With the increasing awareness of the importance of using sunscreens to prevent sunburns, skin cancer, hyperpigmentation disorders and skin aging, demand for sunscreen formulations has increased, creating an opportunity for manufacturers of dermocosmetics to develop good-quality skincare products that are effective, safe and aesthetically appealing to meet the consumer requirements. The science of photoprotection has also developed better scientific knowledge and technologies.^31^, ^33^ Topical photoprotection must follow strict quality parameters, including broad-spectrum and photostable filtering systems, a final formula that offers efficacy, safety and does not affect the environment, and, finally, a texture that forms a homogeneous and stable film on the skin, with a sensory characteristic that favors patient adherence.

Dermatologists should value health campaigns, such as December Orange - a Brazilian initiative by the Brazilian Society of Dermatology to raise awareness about skin cancer and guide patients on photoprotective measures, including responsible and skilled prescription on the application of adequate sunscreens.^2^

## Financial support

L’Oréal Brasil provided funds for the meeting of the authors’ group.

## Authors’ contribution

Flavia Alvim Sant Anna Addor: Approval of the final version of the manuscript; design and planning of the study; collection, analysis, and interpretation of data; effective participation in research orientation; critical review of the literature; critical review of the manuscript.

Hélio Miot: Approval of the final version of the manuscript; design and planning of the study; drafting and editing of the manuscript; critical review of the literature; critical review of the manuscript.

Carolina Reato Marçon: Approval of the final version of the manuscript; design and planning of the study; drafting and editing of the manuscript; critical review of the literature; critical review of the manuscript.

Elimar Gomes: Approval of the final version of the manuscript; design and planning of the study; drafting and editing of the manuscript; critical review of the literature; critical review of the manuscript.

Omar Lupi: Approval of the final version of the manuscript; design and planning of the study; drafting and editing of the manuscript; critical review of the literature; critical review of the manuscript.

Carlos Baptista Barcaui: Drafting and editing of the manuscript; critical review of the literature; critical review of the manuscript.

## Conflicts of interest

The authors are consultants at L’Oreal Brasil and received fees for discussing this research.
